# Mode of bacterial killing affects the inflammatory response and associated organ dysfunctions in a porcine *E. coli* intensive care sepsis model

**DOI:** 10.1186/s13054-020-03303-9

**Published:** 2020-11-14

**Authors:** Paul Skorup, Lisa Maudsdotter, Miklós Lipcsey, Anders Larsson, Jan Sjölin

**Affiliations:** 1 Department of Medical Sciences, Section of Infectious Diseases, Uppsala University, 751 85 Uppsala, SE Sweden; 2 Department of Molecular Biosciences, The Wenner-Gren Institute, Stockholm University, Stockholm, Sweden; 3 Department of Surgical Sciences, Hedenstierna Laboratory, Anesthesiology & Intensive Care, Uppsala University, Uppsala, Sweden; 4 Department of Medical Sciences, Section of Clinical Chemistry, Uppsala University, Uppsala, Sweden

**Keywords:** Antibiotics, Bacteria, Sepsis, Cytokines, Inflammation, Porcine

## Abstract

**Background:**

Sepsis is often treated with penicillin-binding protein 3 (PBP-3) acting β-lactam antibiotics, such as piperacillin-tazobactam, cefotaxime, and meropenem. They cause considerable bacterial structural changes and have in vitro been associated with an increased inflammatory response. In a clinically relevant large animal sepsis model, our primary aim was to investigate whether bacteria killed by a PBP-3-active antibiotic has a greater effect on the early inflammatory response and organ dysfunction compared with corresponding amounts of live or heat-killed bacteria. A secondary aim was to determine whether the addition of an aminoglycoside could mitigate the cefuroxime-induced response.

**Method:**

Killed or live *Escherichia coli* were administrated as a 3-h infusion to 16 healthy pigs in a prospective, randomized controlled interventional experimental study. Cefuroxime was chosen as the PBP-3-active antibiotic and tobramycin represented the aminoglycosides. The animals were randomized to receive (I) bacteria killed by cefuroxime, (II) live bacteria, (III) bacteria killed by heat, or (IV) bacteria killed by the combination of cefuroxime and tobramycin. Plasma endotoxin, tumor necrosis factor alpha, interleukin-6, interleukin-10, leukocytes, and organ function were recorded at the start of the experiment and then hourly for 6 h.

**Results:**

Differences in dynamics of concentration over time between the four treatment groups were found for the three cytokines (*p* < 0.001). Animals receiving cefuroxime-killed bacteria demonstrated higher responses than those receiving live (*p* < 0.05) or heat-killed bacteria (*p* < 0.01). The addition of tobramycin reduced the cefuroxime-induced responses (*p* < 0.001). The cytokine responses were associated with leucocyte activation that was further associated with pulmonary dysfunction and increases in lactate (*p* < 0.01).

**Conclusions:**

In comparison with live or heat-killed bacteria, bacteria killed by a PBP-3-active antibiotic induced an increased inflammatory response that appears to be associated with deteriorated organ and cellular function. The addition of an aminoglycoside to the PBP-3-active antibiotic reduced that response.

## Background

Sepsis is a clinical syndrome of potentially life-threatening organ dysfunction caused by a dysregulated host response to an infection. This syndrome is the most common cause of mortality in critically ill patients [1, 2]. In models of experimental Gram-negative sepsis, the inflammatory response is enhanced after administration of antibiotics [3–5]. Similarly, a recent clinical study reported impairment within hours after antibiotic administration in a large proportion of intensive care patients with sepsis [6]. The most common explanation for this phenomenon has been antibiotic-induced endotoxin release during the killing process, an event that has been extensively demonstrated in vitro [7–9]. Still, evidence of antibiotic-induced endotoxin release in patients is limited and conflicting [10–12]. In fact, in a recent study using a large animal Gram-negative sepsis model antibiotic-induced inflammation and organ impairment were observed but without any notable differences in plasma endotoxin concentrations compared with control animals [5]. If variation in endotoxin levels after antibiotic treatment does not cause the differences in activation of the inflammatory response and clinical impairment in vivo, it may be hypothesized that other mechanisms such as antibiotic-induced structural alterations of the bacteria [13] may play an important role.

Antibiotics with affinity to penicillin-binding protein 3 (PBP-3), such as piperacillin-tazobactam, cefotaxime, and meropenem, are commonly used for the treatment of sepsis [14, 15]. PBP-3-acting antibiotics result in marked bacterial structural changes [7, 13, 16, 17] and in vitro have been associated with an increase in the inflammatory response [18, 19]. Using our large animal intensive care Gram-negative sepsis model [5, 20], the primary aim of the present study was to explore the in vivo effect of ex vivo antibiotic-induced structural changes and whether bacteria killed by a PBP-3-active antibiotic affect the early inflammatory response and organ dysfunction to a greater extent than a corresponding amount of live or heat-killed bacteria.

The recommendation by the Surviving Sepsis Campaign to start antibiotic treatment with two antibiotics with different modes of action [21] has been questioned by the Infectious Diseases Society of America (IDSA) Sepsis Task Force. One of the questions of interest here pertains to whether two antibiotics arrest inflammation more rapidly and effectively than one [22, 23]. A reduced effect on the inflammatory response by the addition of aminoglycosides to β-lactam antibiotics has only been demonstrated in vitro. In these studies, the underlying mechanism was believed to be a reduction of endotoxin release during the killing process [18, 19]. Therefore, a secondary aim was to study the in vivo effect on the inflammatory response of bacteria killed by a β-lactam-aminoglycoside combination in the absence of the killing-induced endotoxin liberation.

## Methods

### Animals and ethic statements

Sixteen clinically healthy landrace-breed piglets of both sexes were included. The study was approved by the Animal Ethics Committee in Uppsala, Sweden (permit no. C155/14), and all animals were handled in compliance with the Guide for the Care and Use of Laboratory Animals. Animals had access to water and food ad libitum until 1 h before the start of the experiment.

### Anesthesia and preparations

The anesthetization preparation, maintaining of anesthesia, and ventilation of animals were performed as in previously described procedures [5, 20]. An additional file describes this process in detail (Additional file 1). Before the experimental start, the animals were allowed a 40-min stabilization time. The intensive care provided a treatment protocol to maintain ventilation, anesthesia, and circulation within preset limits, given in Additional file 2.

### Organism

The *Escherichia coli* (*E. coli*) strain B09-11822 employed in this experiment (O-rough:K1:H7 Statens Seruminstitut, Copenhagen, Denmark) is a serum-resistant clinical isolate originally obtained from a patient with bloodstream infection [20]. According to the minimal inhibitory concentration test (Etest®; Biodisk, Solna, Sweden), this strain is sensitive to cefuroxime (4 μg × mL^−1^) and tobramycin (0.5 μg × mL^−1^).

### Antibiotics

Because cefuroxime (Zinacef®, GlaxoSmithKline, Solna, Sweden) and tobramycin (Nebcina®, Meda, Solna, Sweden) have been used in previous in vivo studies on bacterial killing and endotoxin release using our model [5, 20], these antibiotics were allowed to represent PBP-3-active β-lactam antibiotics and aminoglycosides in the present study.

Furthermore, in the context of experimental antibiotic-induced endotoxin release, cefuroxime has been a representative for PBP-3-active β-lactam antibiotics and tobramycin for aminoglycosides in a large number of in vitro studies [7, 9, 17, 24].

### Experimental design

The study was prospective, parallel-grouped with animals randomized through the sealed envelope technique into four treatment groups (I) bacteria killed by cefuroxime, (II) live bacteria, (III) bacteria killed by heat, and (IV) bacteria killed by the combination of cefuroxime and tobramycin. Each group was comprised of four animals. By killing the bacteria ex vivo and with the removal of the supernatant, the effect of antibiotic-induced structural changes could be studied without the influence of the endotoxin that is liberated during the killing process [7, 9, 24].

### Killing of bacteria ex vivo before the animal experiment

The ex vivo pre-exposures are described in detail in an additional file (Additional file 3). Briefly, in group I (killed by cefuroxime), a bacterial stem solution was pre-exposed to cefuroxime for 4 h. After centrifugation, the fluid containing the liberated endotoxin during the killing process was removed and only the pellet of bacteria was resuspended in saline to the original concentration and then administrated to the animals.

In group II (live bacteria), the stem solution was infused into the animals without pre-exposure.

In group III (killed by heat), the bacterial stem solution was pre-exposed to 95 °C for 10 min and, after having been cooled to room temperature, administrated to the animals.

In group IV (killed by cefuroxime + tobramycin), the bacterial stem solution was pre-exposed to cefuroxime in combination with tobramycin. To reduce the difference in the number of live bacteria in the infusate from that in group I, the exposure time was reduced to 1 h. After that, the bacteria were treated in the same way as in group I.

Bacterial count before and after the pre-exposures was performed. A light microscope (× 1000) was used to inspect the morphologic structures of the bacteria after pre-exposure in comparison with those of the live bacteria.

### Animal experiment

**Fig. 1:**
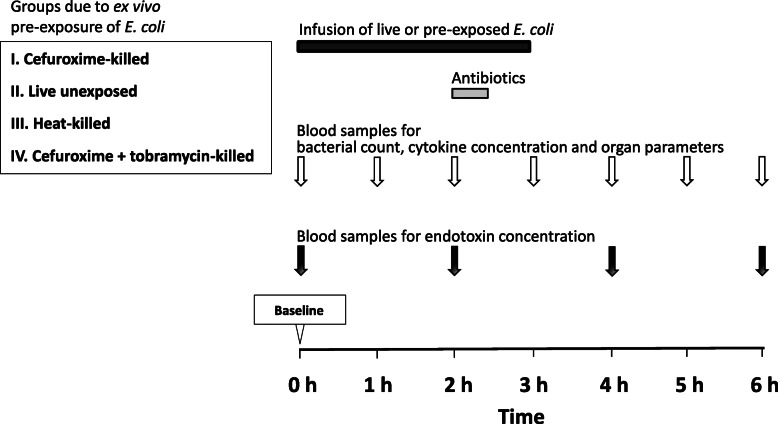
The experimental protocol. The black bar represents an infusion of live or killed bacteria and the gray bar constitutes antibiotics given to eradicate eventual remaining live bacteria in groups I, III, and IV. White arrows indicate time of sampling for bacterial quantification, cytokine concentrations, and organ function parameters. Black arrows designate time of sampling for endotoxin concentrations

The experimental protocol is depicted in Fig. 1. The final bacterial solutions were infused intravenously at a constant rate of 8.33 mL/h starting from baseline and continuing for 3 h. To kill the fraction of eventual remaining live bacteria, antibiotics were provided as 20-min infusions 2 h after baseline as follows: group I and III received 50 mL saline plus 750 mg cefuroxime in 50 mL saline, group II received only 100 mL saline, and animals in group IV were administered 750 mg cefuroxime in 50 mL saline and an additional 7 mg × kg^−1^ tobramycin in 50 mL saline*.* Blood samples were collected at baseline and then at hourly intervals to determine the inflammatory response and cellular and organ function. Levels of endotoxin were measured at 0 h, 2 h, 4 h, and 6 h. Inflammation was evaluated hourly by arterial concentrations of tumor necrosis factor alpha (TNF-α), interleukin-6 (IL-6), interleukin-10 (IL-10), leukocytes, and hemoglobin, while cellular dysfunction was assessed by arterial plasma lactate. Circulation was monitored continuously and recorded hourly by mean arterial pressure (MAP) and left ventricular stroke work index (LVSWI). Organ function was recorded hourly by plasma platelet count, arterial partial pressure of oxygen/inspired fraction of oxygen (PaO_2_/FiO_2_), static pulmonary compliance, plasma creatinine, and urinary output. The animals remained anesthetized until sacrifice, which was done by an injection of potassium chloride at 6 h. In the model used, the effect on inflammatory response, cellular function, circulation, and organ dysfunction of saline alone during 6 h has been shown to be negligible [25, 26].

### Analyses

The endotoxin analysis was performed by the Limulus amoebocyte lysate assay and cytokine analysis by the porcine-specific enzyme-linked immunosorbent assay. Details of the methodological analyses of the blood tests and monitoring of the organ parameters are presented in an additional file (Additional file 4).

### Statistics

The primary endpoint was to study whether the dynamics in concentration of cytokines (TNF-α, IL-6, IL-10) differed between animals receiving bacteria killed by cefuroxime and those receiving live or heat-killed bacteria and secondary endpoint whether the inflammatory response was reduced after the addition of tobramycin. Comparing cytokine peak values with four animals in each group, a power of 0.8, a two-sided α-error of 0.05, and a standard deviation of 15%, the detectable difference was at least 30%. For non-cytokine values, the change from baseline was calculated. To evaluate differences between the treatment groups, an analysis of variance (ANOVA) for repeated measures was performed using the group-by-time interaction term. If the interaction demonstrated significance when all four treatment groups were included, additional ANOVAs were conducted to test differences between the treatment groups. Cytokine peak values were compared by one-way ANOVA. In the correlation analyses between the inflammatory response and changes in cellular and organ dysfunction at 3 h, Pearson’s correlation coefficient was calculated, except for creatinine, in which the Spearman rank correlation was applied.

IL-6, TNF-α, and IL-10 concentrations are log-normally distributed [25] and thus these values were logarithmically transformed. Normally distributed data are expressed as mean ± SD. Non-normally distributed data are presented as median (range) with group comparisons performed by the Kruskal-Wallis test. Differences in provided amounts of norepinephrine were compared using the Mann-Whitney *U* test. A *p* value of < 0.05 was considered significant. All calculations were performed using Statistica™ (v13.2, StatSoft, Tulsa, OK, USA).

## Results

All animals developed signs of either sepsis or septic shock after initiation of the bacterial infusion as manifested by decreases in MAP, LVSWI, platelets, PaO_2_/FiO_2_, and pulmonary compliance and increases in lactate, hemoglobin, and plasma creatinine (Table 1). Bodyweight, dose of infused bacteria, concentrations of remaining live *E. coli* in the infusate, and blood bacterial count in vivo are summarized in Table 2.

**Table 1:** Parameters of inflammation, circulation, and organ function in animals treated with either bacteria killed by cefuroxime alone (*n* = 4), live bacteria (*n* = 4), bacteria killed by heat (*n* = 4), or by a combination of cefuroxime + tobramycin (*n* = 4)

.	Baseline before challenge						
	0 h	1 h	2 h	3 h	4 h	5 h	6 h
**Inflammation**							
**Leucocyte count** (10^9^ × L^−1^), *p* all groups < 0.001; *p* cefur vs live NS; *p* cefur vs heat < 0.001; *p* cefur vs comb < 0.001							
Cefuroxime	12.0 ± 2.9	3.7 ± 2.1	3.7 ± 1.7	3.5 ± 2.2	4.7 ± 2.5	5.3 ± 2.4	6.7 ± 2.1
Live bacteria	10.6 ± 6.3	5.3 ± 2.0	5.8 ± 1.3	7.3 ± 4.3	6.2 ± 1.3	5.9 ± 1.0	5.7 ± 1.1
Heat	9.2 ± 3.0	5.8 ± 1.1	4.3 ± 1.7	4.9 ± 1.0	5.6 ± 1.1	7.2 ± 1.7	10.5 ± 2.4
Cefuroxime + tobramycin	12.1 ± 3.6	6.9 ± 1.9	12.5 ± 4.2	12.4 ± 3.2	11.6 ± 2.2	11.1 ± 2.0	12.8 ± 2.5
**Hemoglobin** (g × L^−1^), *p* all groups 0.016; *p* cefur vs live NS; *p* cefur vs heat NS; *p* cefur vs comb < 0.01							
Cefuroxime	74 ± 5	94 ± 9	100 ± 7	105 ± 12	106 ± 10	110 ± 14	109 ± 13
Live bacteria	72 ± 3	82 ± 11	87 ± 14	95 ± 18	99 ± 13	95 ± 10	93 ± 7
Heat	78 ± 8	96 ± 4	105 ± 6	108 ± 1	108 ± 6	104 ± 9	99 ± 5
Cefuroxime + tobramycin	73 ± 4	83 ± 5	84 ± 7	93 ± 10	93 ± 11	89 ± 9	86 ± 8
**Circulation**							
**Mean arterial pressure** (mm Hg), *p* all groups NS							
Cefuroxime	95 ± 11	83 ± 13	86 ± 19	80 ± 23	80 ± 24	82 ± 20	67 ± 20
Live bacteria	96 ± 11	78 ± 15	87 ± 11	88 ± 7	87 ± 10	88 ± 17	84 ± 18
Heat	93 ± 13	92 ± 11	95 ± 21	82 ± 21	85 ± 23	75 ± 14	73 ± 10
Cefuroxime + tobramycin	100 ± 9	100 ± 6	104 ± 6	113 ± 5	113 ± 9	104 ± 15	92 ± 18
**LVSWI** (g × m × m^−2^), *p* all groups NS							
Cefuroxime	53 ± 1	31 ± 12	30 ± 17	20 ± 12	19 ± 10	19 ± 9	14 ± 8
Live bacteria	56 ± 5	33 ± 8	34 ± 4	29 ± 7	26 ± 8	26 ± 6	25 ± 5
Heat	57 ± 15	42 ± 5	34 ± 13	20 ± 10	24 ± 13	23 ± 7	21 ± 6
Cefuroxime + tobramycin	59 ± 13	46 ± 15	40 ± 12	38 ± 12	43 ± 7	40 ± 7	35 ± 7
**Plasma lactate** (mmol × L^−1^), *p* all groups NS							
Cefuroxime	1.7 ± 0.8	3.2 ± 2.2	3.4 ± 1.8	3.3 ± 1.7	3.3 ± 1.4	2.6 ± 1.3	2.2 ± 1.5
Live bacteria	1.2 ± 0.8	1.8 ± 0.9	1.5 ± 0.5	1.8 ± 0.7	1.6 ± 0.6	1.3 ± 0.2	1.1 ± 0.1
Heat	1.1 ± 0.2	1.4 ± 0.2	2.1 ± 0.7	2.3 ± 0.5	2.0 ± 1.1	1.6 ± 1.0	1.4 ± 0.9
Cefuroxime + tobramycin	1.2 ± 0.1	1.6 ± 0.2	1.3 ± 0.2	1.6 ± 0.3	1.5 ± 0.3	1.1 ± 0.3	0.9 ± 0.1
**Organ function**							
**Platelet count** (10^9^ × L^−1^), *p* all groups NS							
Cefuroxime	343 ± 117	366 ± 147	345 ± 135	258 ± 125	217 ± 165	237 ± 145	248 ± 152
Live bacteria	314 ± 68	365 ± 57	360 ± 78	289 ± 85	304 ± 104	288 ± 97	279 ± 99
Heat	259 ± 56	281 ± 35	212 ± 25	162 ± 38	194 ± 35	208 ± 26	218 ± 28
Cefuroxime + tobramycin	286 ± 53	315 ± 48	300 ± 67	280 ± 59	283 ± 53	286 ± 53	282 ± 64
**PaO**_**2**_**/FiO**_**2**_ (kPa), *p* all groups NS							
Cefuroxime	59 ± 10	47 ± 13	38 ± 16	25 ± 3	34 ± 16	38 ± 18	35 ± 16
Live bacteria	58 ± 10	53 ± 18	45 ± 14	32 ± 23	31 ± 24	36 ± 19	34 ± 18
Heat	60 ± 4	54 ± 12	37 ± 15	32 ± 17	32 ± 14	32 ± 9	32 ± 8
Cefuroxime + tobramycin	60 ± 9	58 ± 10	53 ± 10	45 ± 13	36 ± 14	36 ± 6	34 ± 6
**Static pulmonary compliance** (mL × cmH_2_O^−1^), *p* all groups NS							
Cefuroxime	24 ± 7	20 ± 5	19 ± 7	15 ± 3	15 ± 5	17 ± 4	16 ± 3
Live bacteria	25 ± 5	20 ± 4	18 ± 5	16 ± 5	16 ± 5	16 ± 5	16 ± 3
Heat	21 ± 3	18 ± 3	15 ± 4	15 ± 1	16 ± 1	16 ± 1	16 ± 1
Cefuroxime + tobramycin	23 ± 2	21 ± 2	21 ± 2	19 ± 2	19 ± 1	18 ± 1	18 ± 1
**Plasma creatinine** (μmol × L^−1^) *p* KW all groups 3 h NS; *p* KW all groups 6 h NS							
Cefuroxime	85 (71–90)	78 (68–88)	76 (65–83)	77 (62–90)	91 (58–112)	101 (59–138)	120 (60–159)
Live bacteria	66 (61–121)	64 (61–119)	63 (59–117)	64 (58–110)	66 (60–104)	73 (63–91)	79 (68–90)
Heat	67 (60–70)	67 (61–69)	62 (59–67)	69 (64–74)	75 (65–92)	81 (69–95)	88 (74–95)
Cefuroxime + tobramycin	77 (65–84)	76 (63–81)	74 (62–79)	72 (61–78)	72 (64–83)	77 (69–85)	79 (60–88)
**Urine output** (ml × h^−1^) *p* KW all groups 3 h NS; *p* KW all groups 6 h NS							
Cefuroxime	55 (44–600)	160 (65–480)	84 (65–95)	59 (11–160)	28 (6–60)	16 (10–33)	16 (0–35)
Live bacteria	110 (16–120)	310 (70–490)	65 (45–150)	70 (33–80)	46 (25–70)	35 (17–65)	27 (5–38)
Heat	99 (53–216)	260 (95–500)	183 (60–280)	140 (17–280)	70 (1–190)	67 (0–280)	40 (0–1)
Cefuroxime + tobramycin	43 (16–88)	145 (75–180)	98 (55–210)	78 (70–85)	80 (70–180)	62 (22–95)	83 (70–240)

*NS* not significant, *LVSWI* left ventricular stroke work index, *PaO*_*2*_*/FiO*_*2*_ oxygen fraction ratio, *KW* Kruskal-Wallis test

Mean ± SD or median (range). *p* values are the result of the group-by-time interaction in the repeated-measures ANOVA (0–6 h) or the Kruskal-Wallis test for non-parametric parameters at 3 h and 6 h

**Table 2:** Bodyweight, dose of infused bacteria, and in vivo blood bacterial counts

Variable		Cefuroxime	Live *E. coli*	Heat	Cefuroxime + tobramycin
Bodyweight (kg)		26.0 ± 2.0	24.7 ± 1.4	26.6 ± 1.5	25.5 ± 2.7
Infused bacteria, total dose (log_10_ CFU)		8.78 ± 0.06	8.78 ± 0.07^a^	8.71 ± 0.05	8.74 ± 0.05
Infused bacteria, live bacterial dose (log_10_ CFU)		5.50 ± 0.40	8.78 ± 0.07	0.85 ± 1.70	2.39 ± 1.70
Blood bacterial count in vivo (log_10_ CFU × ml^−1^)	0 h	< 0.5	< 0.5	< 0.5	< 0.5
	1 h	< 0.5	2.83 ± 0.38	< 0.5	< 0.5
	2 h	< 0.5	3.18 ± 0.48	< 0.5	< 0.5
	3 h	< 0.5	3.30 ± 0.53	< 0.5	< 0.5
	4–6 h	< 0.5	< 0.5	< 0.5	< 0.5

Mean ± SD; *CFU* colony-forming unit

^a^These bacteria were not intended to be killed

There were no differences in bacterial dose or animal weight between the treatment groups. A small fraction of live bacteria (< 0.1%) remained in the infusates exposed to heat or antibiotics but this did not result in positive blood bacterial count in vivo.

### TNF-α, IL-6, and IL-10 dynamics

**Fig. 2:**
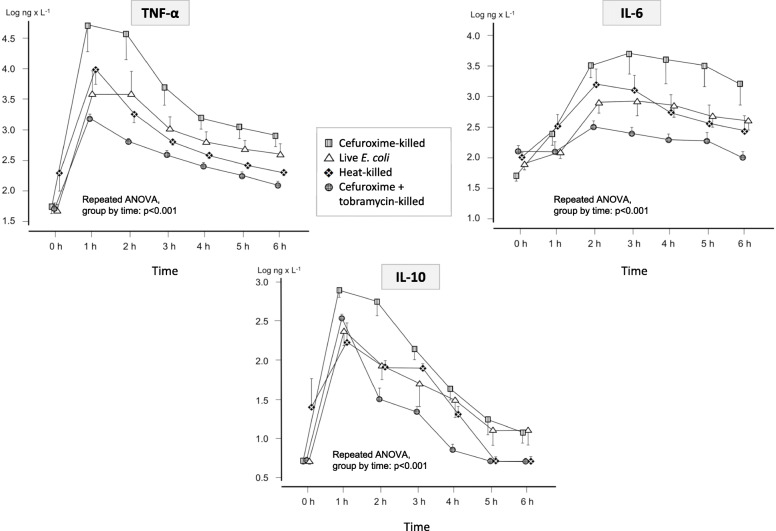
Dynamics in concentration of TNF-α, IL-6, and IL-10 in animals during and after administration of killed or live *E. coli* in the following treatment groups (*n* = 4 in each group): (I) bacteria killed by cefuroxime alone, (II) live bacteria, (III) bacteria killed by heat, and (IV) bacteria killed by the combination of cefuroxime and tobramycin. Data are shown as mean ± SE. *p* values are the results of the group-by-time interaction in the repeated-measures ANOVA after considering all time points

Concentrations of TNF-α, IL-6, and IL-10 are displayed in Fig. 2. At baseline, plasma TNF-α, IL-6, and IL-10 levels were close to the detection limit in all animals, increasing markedly during the study. Plasma TNF-α and IL-10 reached peak concentrations at 1 h and IL-6 at 3 h. Thereafter, the concentrations continuously declined. Differences in dynamics of concentration over time between the four treatment groups were found for the three cytokines (all, *p* < 0.001). Bacteria pre-exposed to cefuroxime resulted in higher cytokine values over time for all three cytokines than live bacteria (all, *p* < 0.05) or bacteria pre-exposed to heat (all, *p* < 0.01). The addition of tobramycin notably reduced the cefuroxime-induced cytokine response (all, *p* < 0.001).

Cytokine peak values in the cefuroxime group were higher than in the combination group (all, *p* < 0.05). However, in contrast to animals administered live or heat-killed bacteria, the differences did not reach statistical significance.

### Inflammation, circulation, and organ dysfunction

Decreases in leukocyte count, which reflect leukocyte activation [27, 28], and increases in hemoglobin, which reflect an increase in capillary permeability [29], differed significantly between the treatment groups, with the most prominent changes in the cefuroxime group and the most discrete changes in the combination group (Table 1).

The total dose of norepinephrine, provided as per the intensive care protocol, was higher in the cefuroxime group with a median dose of 2440 μg (range 60–4980), which was more than that given to animals administered live bacteria (median dose 0 μg, range 0–60) or bacteria killed by the combination antibiotic treatment that were not given any norepinephrine at all (both *p* < 0.05). For LVSWI, plasma lactate, and organ function, there were more pronounced changes in the group pre-exposed to cefuroxime, although these differences were not statistically significant (Table 1). Other intensive care adjustments, such as increased FiO_2_ or extra fluid boluses, were carried out in accordance with the treatment protocol without significant differences between the groups.

As shown in Table 3, the peak TNF-α and IL-6 values correlated with changes at 3 h in lactate, platelets, and PaO_2_/FiO_2_. Similarly, the leukocyte response correlated with changes at 3 h in lactate and PaO_2_/FiO_2_. For changes in creatinine, these correlations were stronger at 6 h: 0.86 for TNF-α, 0.81 for IL-6, and − 0.64 for leukocyte peak responses.

**Table 3:** Correlations between cytokines, leukocyte response, and changes in hemoglobin, lactate, and organ function variables

Variable	Log IL-6_3h_	Log IL-10_1h_	Leukocytes, 3–0 h	Hemoglobin, 3–0 h	LVSWI, 3–0 h	Lactate, 3–0 h	Platelets, 3–0 h	Stat. pulm. Compl., 3–0 h	PaO_2_/FiO_2_, 3–0 h	Creatinine, 3–0 h
Log TNF-α_1h_	0.87***	0.71**	− 0.79***	0.32	− 0.57*	0.88***	− 0.63**	− 0.19	− 0.62*	0.44
Log IL-6_3h_	–	0.47	− 0.71**	0.34	− 0.61*	0.77***	− 0.65**	− 0.29	− 0.78***	0.39
Log IL-10_1h_	–	–	− 0.63**	0.14	− 0.27	0.55*	− 0.11	− 0.24	− 0.43	− 0.05
Leukocytes_3-0h_	–	–	–	− 0.40	0.34	− 0.70**	0.38	0.49	0.77***	− 0.07

*LVSWI* left ventricular stroke work index, *Stat. pulm. Compl.* static pulmonary compliance

Pearson’s correlation test was used, except in the correlations for creatinine in which Spearman’s rank correlation test was calculated

*p < 0.05; **p < 0.01; ***p < 0.001

### Morphologic visualization of bacteria in the infused solutions and endotoxin levels

Microscopic investigation of the final infused solutions containing live bacteria, bacteria killed by heat, or the combination of cefuroxime + tobramycin visualized bacteria that could not be differentiated in quantity or morphology from each other. In contrast, solutions with bacteria killed by cefuroxime alone demonstrated elongated thread-like bacterial filaments without visible damage to the membranes.

Endotoxin concentrations in the four treatment groups are listed in Table 4. The peak endotoxin concentrations at 2 h correlated with peak TNF-α, Il-6, and IL-10 values and leukocytes response at 3 h with *r* values of 0.88, 0.85, 0.42, and − 0.61, respectively (all, *p* < 0.05).

**Table 4:** Endotoxin levels at different time points

Treatment	Baseline, before challenge			
	0 h	2 h	4 h	6 h
Cefuroxime	1.53 ± 0.20	2.98 ± 0.72	2.27 ± 0.50	1.98 ± 0.42
Live bacteria	1.57 ± 0.28	2.67 ± 0.39	2.21 ± 0.30	1.82 ± 0.09
Heat	1.83 ± 0.33	2.76 ± 0.12	2.09 ± 0.26	1.77 ± 0.21
Cefuroxime + tobramycin	1.50 ± 0.20	2.08 ± 0.19	1.52 ± 0.15	1.50 ± 0.12

Mean ± SD; Log EU × L^−1^

## Discussion

Comparing with previously used rodent models [3, 4], the sepsis model used in the present study takes advantage of the porcine similarities to the human inflammatory response [30]. In addition, the model was further developed to resemble the situation for patients with sepsis admitted to an intensive care unit, where therapeutic actions such as sedation, mechanical ventilation, and vasopressors may modify the inflammatory response [20, 31, 32]. Despite the mitigating effects of these intensive care measures and in the absence of the free endotoxin that has been shown to be liberated during the antibiotic-induced killing process in vitro [7], bacteria killed by the PBP-3-active antibiotic cefuroxime resulted in increased TNF-α and IL-6 responses compared with those caused by live, untreated bacteria. There were also significant cytokine-associated differences in leukocyte activation and capillary leakage. Although significant correlations do not establish causal relationships, the differences observed in the inflammatory response might be further linked to changes in cellular metabolism and organ function, thus lending support to the non-significant changes in these parameters seen in Table 1.

Because one dose of PBP-3-active antibiotics does not kill all bacteria [5, 7], some remaining live bacteria were also included in the infusate. Because of the low number of live bacteria relative to the number of killed bacteria and because of the extra dose of antibiotics administered after 2 h, it is unlikely that these live bacteria affected the cefuroxime-induced response. Moreover, a mixture of killed and live bacteria is an obvious phenomenon in vivo. Animals that were given cefuroxime-killed bacteria also demonstrated a higher inflammatory response than animals given heat-killed bacteria, which resulted in a response similar to that caused by live bacteria. This finding indicates that the cefuroxime-induced inflammatory increase was not elicited simply because the bacteria were not alive.

IL-10, an early marker associated with the initiation of anti-inflammatory reactions, increased in parallel with TNF-α and IL-6, supporting initial activation of both pro- and anti-inflammatory responses in this model, which is similar to early clinical responses shown in intensive care patients with sepsis [33, 34]. IL-10 was consistently higher in the cefuroxime group as compared with the other groups.

We hypothesize that the bacterial morphological appearance with elongation and formation of filamentous forms associated with PBP-3-activity described previously [16, 17, 35] and seen in the present experiment plays an important role in the induction of inflammation and organ dysfunction. Larger bacterial areas due to elongation most certainly increase the amount of pathogen-associated molecular patterns that can bind with extracellular domains on host inflammatory cells. Because the supernatants after treatment with antibiotics or heat were removed before infusion into the animals, the free endotoxin in this study probably originated from killed bacteria in vivo during the further process of fragmentation. The correlations between free endotoxin peak levels and TNF-α, IL-6, and leukocyte responses suggest that liberated endotoxin might contribute to the inflammatory response. However, a study on the porcine dose-response of cytokines to endotoxin [25] indicates that the limited concentration differences between the treatment groups would only result in a minor modification of the cytokine response.

Live bacteria are quickly eliminated from the circulation because of an effective immune system and the process of bacterial killing takes mainly place in the spleen and liver [20]. Using this intensive care model in a previous study, animals with live *E. coli* sepsis treated with cefuroxime demonstrated a more pronounced cytokine increase, leukocyte activation, and organ dysfunction than control animals. These events occurred without any increase in plasma free endotoxin [5]. The increased inflammatory response might have been the result of the killing process with or without local endotoxin liberation in the liver and the spleen or the result of the antibiotic-killed bacteria per se. The present results support the findings of that study and further demonstrate that killed bacteria alone can elicit the inflammatory response and that the mechanism of the preceding killing results in inflammatory responses of different magnitudes.

Our results support Mock et al.’s clinical findings in which patients with Gram-negative sepsis treated with PBP-3-active antibiotics showed higher mortality than patients treated with antibiotics with little or no PBP-3-activity [36]. Randomized clinical studies comparing the effect of a PBP-3-active antibiotic with a non-PBP-3-active antibiotic have shown no or only trends towards differences in endotoxin levels, cytokines, and outcomes [37–40]. However, considering the mild severity of sepsis and the low number of patients included in these studies, the chance of detecting a difference was minimal. A randomized clinical study specifically on patients with septic shock would be of value but has not yet been performed, probably because of the inherent difficulties in conducting such a study. Nevertheless, the phenomenon of an antibiotic-induced inflammatory response and deterioration is in agreement with Mignon et al.’s study on intensive care patients with sepsis in which deterioration occurred in almost half of the patients within 4 h after the start of antibiotic treatment [6].

As has previously been shown in vitro [18, 19], this large animal study demonstrated that the addition of tobramycin reduced the cefuroxime-induced inflammatory response also in vivo. This effect seems to be mainly mediated by non-endotoxic effects. The morphologic elongation of bacteria seen after single PBP-3-active treatment was absent when tobramycin was added, which might explain this result. However, in agreement with several in vitro studies [7, 9, 24], the levels of endotoxin were lower in the combination group, suggesting that differences in endotoxin levels might have contributed to the reduced inflammatory response in the combination group. In addition to its binding to the ribosome causing mistranslation and misfolded membrane proteins as well as changes in the bacterial surface, the aminoglycoside-induced inhibition of endotoxin synthesis may play a role [13, 41]. These results, in conjunction with the finding of a more rapid killing [20], indicate that the beneficial effects of adding an aminoglycoside to a β-lactam antibiotic found in vitro also are seen in vivo. The more rapid killing and a the decreased inflammatory response demonstrated in this intensive care sepsis model, corresponding to severely septic ICU patients, thus offer possible explanations to Kumar et al.’s clinical findings in which patients treated with the combination of a β-lactam antibiotic and an aminoglycoside demonstrated improved survival in comparison with propensity-matched control patients [42] a clinical finding seen in the most critically ill patients, i.e., those with septic shock [43, 44]. Although a theoretical experimental model, the results from this large animal intensive care model lend some support to the hypothesis that a combination of antibiotics may arrest inflammation more rapidly and effectively than single treatment which was one of the questions raised by IDSA [22, 23] in their criticism of the Surviving Sepsis Campaign’s recommendation to combine antibiotics for the treatment of patients in septic shock [21].

## Conclusions

*E. coli* killed by a PBP-3-active β-lactam antibiotic induces an increased inflammatory response when compared with live or heat-killed bacteria. This effect seems to be mediated mainly by other mechanisms than endotoxin release. The increase in inflammation appears to be associated with deteriorated organ and cellular function. The addition of an aminoglycoside reduces the PBP-3-active antibiotic-induced inflammatory response.

## Supplementary information

**Additional file 1.** Anesthesia, ventilation, preparations and intensive care settings.

**Additional file 2.** Intensive care treatment protocol.

**Additional file 3.** Killing of bacteria ex vivo before the animal experiment, methodology.

**Additional file 4.** Detailed description of blood test analyses and monitoring of organ parameters.

## Data Availability

Data are not collected from a public database. All relevant data are available upon request from the corresponding author.

## Abbreviations

ANOVA: Analysis of variance
CFU: Colony-forming units
*E. coli*: *Escherichia coli*
FiO_2_: Inspired fraction of oxygen
IDSA: Infectious Diseases Society of America
IL: Interleukin
LVSWI: Left ventricular stroke work index
MAP: Mean arterial pressure
PaO_2_: Arterial partial pressure of oxygen
PBP-3: Penicillin-binding protein 3
TNF-α: Tumor necrosis factor alpha
