# Supplementary material for: Mode of bacterial killing affects the inflammatory response and associated organ dysfunctions in a porcine E. coli intensive care sepsis model
Source: Crit Care. 2020 Nov 14;24:646. doi: 10.1186/s13054-020-03303-9 (PMC7666448; doi:10.1186/s13054-020-03303-9)
Supplement: Supplementary file 1 — Additional file 1. Anesthesia, ventilation, preparations and intensive care settings. [file 13054_2020_3303_MOESM1_ESM.docx]

# **Additional file 1:**

# Manuscript title: Mode of bacterial killing affects the inflammatory response and associated organ dysfunctions in a porcine *E. coli* intensive care sepsis model.

## Anesthesia, ventilation, preparations and intensive care settings

All preparations were performed under aseptic conditions. General anesthesia was induced by an intramuscular injection of 6mg x kg^-1^ tiletamine/zolazepam (Zoletil^®^, Reading Laboratories, Carros, France) combined with 2.2 mg x kg^-1^ xylazine (Rompun^®^, Bayer, Germany) and 0.04 mg kg^-1^ atropin (Atropin^®^, NM Pharma, Stockholm, Sweden). The animals were tracheally intubated after an auricular intravenous (i.v.) injection of 20 mg of morphine (Morfin Bioglan^®^, Uppsala, Sweden) and 100 mg of ketamine (Ketaminol^®^, Veterinaria AG, Switzerland). Before baseline, the animals underwent 40 minutes (min) of stabilization after completed preparations. During the experiment, general anesthesia was maintained with a continuous i.v. infusion containing 8 mg x kg^-1^ x h^-1^ sodium pentobarbital (Pentobarbitalnatrium^®^, Apoteket, Umeå, Sweden), 0.26 mg x kg^-1^ x h^-1^ pancuronium bromide (Pavulon^®^, Organon, Oss, The Netherlands) and 0.48 mg x kg^-1^ x h^-1^ morphine (Morfin Bioglan^®^, Uppsala, Sweden) dissolved in a 2.5% glucose solution (Rehydrex^®^, Frenius Kabi, Uppsala, Sweden) at 8 mL x kg^-1^ x h^-1^. Fluid replacement of acetated Ringer’s solution (Ringeracetat®, Frezenius Kabi, Stockholm, Sweden) was administered i.v. at 7 mL x kg^-1^ x h^-1^, resulting in a total fluid administration rate of 15 mL x kg^-1^ x h^-1^during the experiment. The animals were mechanically ventilated using either a Servo I^®^ or a Servo 900C^®^ ventilator (Siemens-Elema, Stockholm, Sweden). Respiratory settings were as follows: volume-controlled mode, inspired fraction of oxygen (FiO_2_) 30%, respiratory rate 25 min^-1^ and positive end-expiratory pressure (PEEP) 5 cm H_2_O. Tidal volume was adjusted during the upstart period to maintain an arterial partial pressure of carbon dioxide (PaCO_2_) of 5.0-5.5 kPa. A bolus of 4% succinylated gelofusine solution (Gelofusin^®^, B. Braun, Meslungen, Germany) 30 mL x kg^-1^ was given 45-60 min before the start of the bacterial infusion.

Catheterization was carried out in an auricular peripheral vein, a superior caval vein, a pulmonary artery (Swan Ganz catheter) and a cervical artery. A vesicostomy was performed and an indwelling catheter introduced into the bladder.
