# Supplementary material for: Mode of bacterial killing affects the inflammatory response and associated organ dysfunctions in a porcine E. coli intensive care sepsis model
Source: Crit Care. 2020 Nov 14;24:646. doi: 10.1186/s13054-020-03303-9 (PMC7666448; doi:10.1186/s13054-020-03303-9)
Supplement: Supplementary file 2 — Additional file 2. Intensive care treatment protocol. [file 13054_2020_3303_MOESM2_ESM.docx]

# Additional file 2:

# Manuscript title: Mode of bacterial killing affects the inflammatory response and associated organ dysfunctions in a porcine *E. coli* intensive care sepsis model.

## Intensive care treatment protocol

Maintenance of the intensive care setting was accomplished through interventions following a treatment protocol to preserve the vital parameters within preset limits according to the Table below.

| **Variable** | **Threshold values for intervention** | **Interventions** |
| --- | --- | --- |
| **PaO_2_** | <10 kPa first time | Increase FiO_2_ to 0.6 |
| **PaO_2_** | <10 kPa thereafter | 1. Increase FiO_2_ to the next level: 0.6🡪0.8🡪1.0  AND  2. Increase PEEP to the next level: 5🡪8🡪10🡪14 cmH_2_0  AND  3. Lung recruitment maneuver ^a^ |
|  |  |  |
|  |  |  |
|  |  |  |
|  |  |  |
| **PaO_2_** | >30 kPa | Decrease FiO_2_ to the next level: 1.0🡪0.8🡪0.6🡪0.3 |
| **MAP and /or CI** | MAP <60 mm Hg and/or CI <2.0 L x min^-2^ x m^-2^ | Start norepinephrine infusion with 0.07 µg x kg^-1^ x min^-1^. If norepinephrine infusion is in progress, increase rate one step: 0.07🡪0.13🡪0.29🡪0.54 µg x kg^-1^ x min^-1^ |
| **MAP** | MAP =MPAP (at <1 hour (h) after baseline) | Single dose of 40 µg norepinephrine i.v. |
| **MAP** | MAP =MPAP (at >1 h after baseline) | 1. Single dose of 20 µg norepinephrine i.v.  AND  2. Start norepinephrine infusion with 0.07 µg x kg^-1^ x min^-1^. If norepinephrine infusion is in progress, increase rate one step: 0.07🡪0.13🡪0.29🡪0.54 µg x kg^-1^ x min^-1^  AND  3. Fluid bolus of 10 mL x kg^-1^ with 4% succinylated gelatin in normal saline  AND  4. Increase FiO_2_ to the next level: 0.3🡪0.6🡪0.8🡪1.0  AND  5. Increase PEEP to the next level: 5🡪8🡪10🡪14 cmH_2_0  AND  6. Lung recruitment maneuver ^a^ |
|  |  |  |
|  |  |  |
|  |  |  |
|  |  |  |
|  |  |  |
|  |  |  |
|  |  |  |
|  |  |  |
|  |  |  |
|  |  |  |
| **MAP** | >100 mm Hg | If norepinephrine infusion is in progress, decrease rate one step: 0.54🡪0.29🡪0.13🡪0.07 µg x kg^-1^ x min^-1^ |

PaO_2_= arterial partial pressure of oxygen, FiO_2_= inspired fraction of oxygen, PEEP= positive end-expiratory pressure, MAP= mean arterial pressure, CI= cardiac index, MPAP= mean pulmonary arterial pressure.

^a^ PEEP was increased stepwise until a peak pressure of 35 cm H_2_O was reached. At that point, an inspiratory hold was performed for 10 seconds. Thereafter, the PEEP was decreased stepwise to the PEEP defined by the protocol. If MAP decreased to the level of the MPAP, the recruitment maneuver was aborted.
