# Supplementary material for: Mode of bacterial killing affects the inflammatory response and associated organ dysfunctions in a porcine E. coli intensive care sepsis model
Source: Crit Care. 2020 Nov 14;24:646. doi: 10.1186/s13054-020-03303-9 (PMC7666448; doi:10.1186/s13054-020-03303-9)
Supplement: Supplementary file 3 — Additional file 3. Killing of bacteria ex vivo before the animal experiment, methodology. [file 13054_2020_3303_MOESM3_ESM.docx]

# Additional file 3:

# Manuscript title: Mode of bacterial killing affects the inflammatory response and associated organ dysfunctions in a porcine *E. coli* intensive care sepsis model.

## Killing of bacteria *ex vivo* before the animal experiment, methodology

The animals were randomized to receive live or killed *E. coli* for 3 h.

The bacteria were harvested 1 day in advance of the experiment and thereafter grown to logarithmic growth phase before the experiment and resuspended in saline resulting in an *E. coli* stem solution of 2.3 x 10^7^ CFU x mL^-1^ with an accepted range of 1.8-2.9 x 10^7^ CFU x mL^-1^ (7.36 + 0.10 log_10_ CFU x mL^-1^). All *ex vivo* pre-exposure procedures with antibiotics and heat were performed using this stem solution. Samples were obtained before and after pre-exposure for bacterial quantification employing serial dilutions, plating in duplicate and the viable count technique after incubation overnight.

#### Antibiotic killing of bacteria

To optimize antibiotic killing of bacteria the bacterial stem solution was diluted 10 times with saline and then treated in an incubator at 37^o^C with either cefuroxime alone at a concentration of 10µg x mL^-1^ for 4 h or cefuroxime at the same concentration in combination with tobramycin at a concentration of 20µg x mL^-1^ for 1 h. These antibiotic concentrations correspond to those observed in clinical practice and levels obtained in our porcine model (1). After antibiotic treatment, the solution was centrifuged, the supernatant removed and the solution resuspended with saline back to the original stem solution concentration. This solution was kept in 3-5^o^C for 16-18 h and then for 1 h in room temperature before administration to the animals.

#### Heat killing of bacteria

Glass tubes containing the *E. coli* stem solution were installed for 10 min on a 95° C electric thermoblock (Gebr. Liebisch, Bielefeld 14, Germany) within the last 30 min before baseline. Thereafter, the bacterial solution was cooled to room temperature for 30 min before infusion.

#### Untreated live bacteria

The *E. coli* stem solution was prepared within the last 60 min before baseline and infused into the animals. The stem solution was analyzed for bacterial quantification as described above. To secure that the bacteria remained in log phase the infusate was replaced hourly.

## Reference:

1. Skorup P, Maudsdotter L, Lipcsey M, Castegren M, Larsson A, Jonsson AB, et al. Beneficial antimicrobial effect of the addition of an aminoglycoside to a beta-lactam antibiotic in an E. coli porcine intensive care severe sepsis model. PLoS One. 2014;9(2):e90441.
