# Supplementary material for: Mode of bacterial killing affects the inflammatory response and associated organ dysfunctions in a porcine E. coli intensive care sepsis model
Source: Crit Care. 2020 Nov 14;24:646. doi: 10.1186/s13054-020-03303-9 (PMC7666448; doi:10.1186/s13054-020-03303-9)
Supplement: Supplementary file 4 — Additional file 4. Detailed description of blood test analyses and monitoring of organ parameters. [file 13054_2020_3303_MOESM4_ESM.docx]

# Additional file 4:

# Manuscript title: Mode of bacterial killing affects the inflammatory response and associated organ dysfunctions in a porcine *E. coli* intensive care sepsis model.

## Detailed description of blood test analyses and monitoring of organ parameters.

Arterial blood samples were analyzed hourly for leucocyte and platelet counts, hemoglobin, lactate, creatinine and blood bacterial count. Mean arterial pressure (MAP), left ventricular stroke work index (LVSWI), oxygen fraction ratio (PaO_2_/FiO_2_), static pulmonary compliance and urinary output were registered hourly.

#### Plasma endotoxin

Plasma samples were collected at 0h, 2h, 4h and 6h in endotoxin-free heparinized tubes, immediately filtered (0.45µm) and, after centrifugation, the supernatants were transferred to endotoxin-free tubes. Aliquots were stored at -70°C pending analysis. Endotoxin analysis was performed with the kinetic chromogenic limulus amoebocyte lysate (LAL) assay (Endochrome-K; Charles River Endosafe, Charleston, SC, USA) (1, 2) using endotoxin-free equipment.

#### Plasma cytokines

Tumor necrosis factor alfa (TNF-α), interleukin-6 (IL-6) and interleukin-10 (IL-10) were determined by a commercial porcine-specific sandwich enzyme-linked immune-sorbent assay (ELISA) in plasma (DY690B [TNF-α] and DY686 [IL-6] R&D Systems, Minneapolis, MN, USA), (KSC0102 [IL-10], Invitrogen, Camarillo, CA, USA). The ELISAs had an intra-assay coefficient of variation (CV) of <5% and a total CV of <10%.

#### Leucocyte activation and platelets

An inverse relation was found between leucocyte count and severity of sepsis in this model (3) in accordance with the trapping of initiated neutrophils in the organs during early dysfunction (4, 5). A decrease in leucocyte count was therefore used as a marker for leucocyte activation. Blood leucocyte and platelet counts were analyzed on a CELL-DYN 4000^®^ system (Abbott Scandinavia AB, Kista, Sweden).

#### Blood bacterial count in vivo

Bacterial count in arterial blood at baseline was determined in triplicate by a viable count procedure after plating 0.1 ml blood on Luria-Bertani agar plates overnight.

#### Organ function

Starting at baseline, MAP and urine production were continuously monitored. LVSWI, static pulmonary compliance and PaO_2_/FiO_2_ were calculated using standard formulas.

Arterial blood gases were analyzed for gas tensions (PaO_2_, PaCO_2_), hemoglobin and saturation using an ABL^®^ 300 gas analyzer (ABL^®^ 800 for three animals) and a Hemoximeter^®^ (Radiometer, Brønhøj, Denmark); for lactate, an i-STAT^®^ 1 system (Abbott Scandinavia, Solna, Sweden). Plasma creatinine was analyzed on an Architect ^®^ Ci8200

analyzer (Abbott Scandinavia, Solna, Sweden).

## References:

1. Nachum R, Shanbrom E. Rapid detection of Gram-negative bacteriuria by Limulus amoebocyte lysate assay. J Clin Microbiol. 1981;13(1):158-62.
2. Lindsay G, Roslansky P, Novitsky T. Single-step, chromogenic Limulus amebocyte lysate assay for endotoxin. J Clin Microbiol. 1989;27(5):947-51.
3. Skorup P, Maudsdotter L, Tano E, Lipcsey M, Castegren M, Larsson A, et al. Dynamics of endotoxin, inflammatory variables, and organ dysfunction after treatment with antibiotics in an Escherichia coli porcine intensive care sepsis model. Crit Care Med. 2018;46(7):e634-e41.
4. Brown K, Brain S, Pearson J, Edgeworth J, Lewis S, Treacher D. Neutrophils in development of multiple organ failure in sepsis. The Lancet. 2006;368(9530):157-69.
5. Aziz M, Jacob A, Yang W-L, Matsuda A, Wang P. Current trends in inflammatory and immunomodulatory mediators in sepsis. J Leukoc Biol. 2013;93(3):329-42.
